# Assessing the feasibility of near-infrared spectroscopy for evaluating physiological exercise thresholds

**DOI:** 10.1038/s41598-025-14920-1

**Published:** 2025-09-29

**Authors:** Simon Iskra, Armin Paravlić

**Affiliations:** 1 Faculty of Sport, University of Ljubljana, Ljubljana, Slovenia; 2 Science and Research Centre Koper, Institute for Kinesiology Research, Koper, Slovenia; 3 Faculty of Sport Studies, Masaryk University, 625 00 Brno, Czech Republic

**Keywords:** NIRS, Ventilatory threshold, Cycling, Gas-exchange, Incremental test, Cardiovascular biology, Metabolism

## Abstract

To examine the feasibility of using near-infrared spectroscopy (NIRS) for physiological threshold detection and whether NIRS-derived parameters differ between highly-trained and less-trained cyclists. Twenty-seven male cyclists were divided into: highly trained endurance cyclists (EA) and recreational cyclists (RA). Participants performed a step-incremental cycling test to exhaustion. Ventilatory thresholds (VT_1_ and VT_2_) were determined using gas-exchange variables. NIRS sensor was placed on the vastus lateralis muscle to identify breakpoints corresponding to ventilatory thresholds. No significant differences were observed between NIRS-derived thresholds, compared to VT_1_ and VT_2_ (F = 1.04–1.33, *p* = 0.26–0.36). Moderate to strong correlations were found between NIRS-derived thresholds and ventilatory thresholds (r = 0.65–0.9, *p* < 0.01). A moderate correlation was found between maximal oxygen uptake and minimal tissue saturation index (TSI) value during the test (r = − 0.411, *p* = 0.037). EA group showed tendency towards lower minimal TSI values compared to RA group (MD = 5.46% TSI, *p* = 0.081). NIRS is a feasible tool for non-invasive assessment of ventilatory thresholds during incremental exercise. TSI, in particular, showed lower variability compared to other NIRS-derived parameters, and may therefore be more suitable for practical applications in sport science. Highly trained athletes demonstrated distinct physiological responses compared to recreational athletes, suggesting enhanced peripheral oxygen extraction.

## Introduction

Graded exercise testing (GXT) is widely used in sports and exercise science to determine relevant metabolic thresholds for optimal training prescription. An array of different physiological parameters can be used to determine metabolic thresholds including blood lactate^1^, ventilatory parameters^2^, electromiography^3^, heart rate^4^, muscle oxygenation^5^ and others.

During GXT, athletes perform a step- or ramp incremental test with graded intensity progression from low to maximal levels, often to exhaustion. Monitoring different parameters during GXT allows for the determination of two breakpoints (e.g., thresholds)^2,4,6^ that can be derived from the aforementioned measurement. In general, these thresholds correspond to two physiological transition points where the cardiorespiratory and metabolic systems adapt in response to increasing exercise intensity. The most common method for determining metabolic thresholds is gas exchange analysis, which involves detecting non-linear increases in oxygen uptake (V̇O_2_), minute ventilation (V̇E), and carbon dioxide production (V̇CO_2_) to determine the first ventilatory threshold (VT_1_) and the second ventilatory threshold (VT_2_)—the latter corresponding to the respiratory compensation point (RCP)^7^. Alternatively, blood lactate measurements can also be used to determine two blood lactate thresholds through different methodological approaches. Although both methods are widely used in exercise testing, gas-exchange measurements necessitate costly equipment and specialized laboratories, whereas blood lactate measurements are somewhat more accessible. However, this approach is invasive and cannot be measured continuously^8^.

Near-infrared spectroscopy (NIRS) is an emerging method for directly measuring metabolic perturbations within working muscles during exercise testing and training. It provides valuable insights into relative changes in oxygenated haemoglobin (O_2_Hb) and deoxygenated haemoglobin (HHb). In addition, an approximation of total haemoglobin (tHb) and tissue saturation index (TSI) can be calculated from NIRS measurements. Various studies have investigated the use of NIRS in both symptomatic and healthy populations with good reproducibility^8,9^. NIRS measurements enable direct insights into muscle oxygen extraction within a working muscle, thus offering an additional insight in muscle physiology during GXT. During the progression of GXT, the muscle becomes increasingly deoxygenated, which is reflected in the response of O_2_Hb, HHb, and consequently TSI, potentially enabling the non-invasive and on-site determination of metabolic thresholds using NIRS.

Numerous studies on threshold determination comparing NIRS and other methods have been published, using different NIRS parameters^10,11^. The intensity during GXT increases, HHb values increase accordingly, reaching a plateau towards the end of GXT. The nature of HHb increase during GXT has been previously explained as a segmented linear^12^ or sigmoidal^10,11^ curve. Through different analytical methods, thresholds identified using HHb levels have been linked with those derived from lactate measurements or ventilatory parameters.

Among other NIRS-derived parameters, TSI has recently been proposed to be more suitable for thresholds determination. TSI is calculated from both O_2_Hb and HHb values^13^ and has been linked to changes in local muscle oxygen extraction and arterio-venous difference during exercise^14^. TSI during GXT exhibits a 4-phase response, characterized by: 1) a slight increase or plateau in the beginning; 2) a linear or exponential decline with increasing exercise intensity; 3) plateau towards the end of GXT; 4) rapid reoxygenation immediately after test cessation, lasting for up to two minutes^12,15^. Due to its predictive pattern, TSI has been associated with lactate and metabolic thresholds. However, considerable heterogeneity persists in breakpoint detection methodologies. Studies have primarily examined associations between TSI and VT_2_^16,17^ while others have extended this to include both VT_1_ and VT_2_ thresholds^13,18^.

In addition to threshold determination, NIRS-derived parameters have been employed to differentiate athletes’ training status. For example, studies utilizing repeated transient arterial occlusions have observed distinct kinetics in NIRS-derived parameters between athletes of varying training backgrounds^19,20^. Similarly, differences in HHb and TSI kinetics have been observed during repeated exercise transitions and during GXT^21,22^. Reinpõld et al.^18^ recently investigated the influence of age and performance in a cohort of older and younger well-trained cyclists with different training experience. Their findings revealed variations in the timing of threshold occurrence and a tendency for older cyclists to exhibit lower minimal TSI values despite lower VO_2max_ levels. This phenomenon may reflect age-related physiological adaptations or the cumulative effect of prolonged training experience.

Although NIRS measurements provide valuable physiological insights into working muscles, several issues persist in the published literature, including inconsistencies in signal processing, threshold detection methodologies used, and populations studied. Whether NIRS-derived parameters can effectively differentiate between athletes of varying training levels remains unclear. Therefore, the aim of this study was twofold: to examine: (a) the feasibility of using different NIRS-derived parameters (HHb and TSI) for physiological threshold detection, and validate them against ventilatory thresholds during incremental cycling exercise, and (b) whether NIRS-derived parameters differ between highly trained and less-trained cyclists. The findings of this study will aid sports scientists and practitioners in better understanding the utility of NIRS measurements for physiological threshold determination and their potential to assess the training status of cyclists.

## Methods

### Participants

The sample consisted of 27 participants, divided into two groups based on their training status. Fourteen prospective male youth cyclists were recruited as highly trained endurance athletes (EA), i.e., Tier 3 and Tier 4 athletes^23^. The inclusion criteria were: (1) national or international prospective athlete categorization—based on the national Olympic committee categorization, (2) age 16–23 years, (3) V̇O_2peak_ > 65 ml.kg^-1^.min^-1^, (4) body fat percentage (BF%) < 20%, 5) < 20 mm adipose tissue thickness (ATT) at the NIRS sensor placement site. Additionally, 13 recreationally active male cyclists were included in the study, representing recreational athletes (RA) group, i.e., Tier 2^23^. Inclusion criteria: (1) 16 up to 35 years of age, (2) V̇O_2peak_ > 50 ml.kg^-1^.min^-1^, (3) BF% < 20, (4) 3) < 20 mm ATT at the measurement site, (5) > 3 times/week cycling training, (6) recreational athlete. All participants were healthy, non-smokers, and without history of using prescribed medication. The testing procedure excluding the addition of NIRS sensor is part of a regular testing procedure used for categorized athletes in our laboratory. All participants were informed of the study design and risks involved with participation in the study. All participants provided informed consent prior to their participation in the study. For participants under the age of 18, additional consent was obtained from a parent or legal guardian. The study was performed in accordance with the Helsinki Declaration and was approved by the ethics committee of Faculty of Sport, University of Ljubljana (ID:033-6-/2024-45).

### Study design

All testing sessions took place in the laboratory of exercise physiology at the Faculty of Sport, University of Ljubljana, under controlled ambient conditions. Participants were instructed to arrive well rested and adequately hydrated. They were advised to abstain from caffeine and alcohol consumption, as well as strenuous exercise for at least 72 h before the test. Additionally, participants were instructed to eat a moderate, carbohydrate-rich meal 2–3 h before arriving at the laboratory. Upon arrival at the laboratory, participants’ body mass and body composition were measured using a bioimpedance device (Biospace InBody 720, South Korea). Body height was measured using an anthropometer (GPM, Siber Hegner & Co., Switzerland). Afterward, participants were instructed to lie on a table while the NIRS sensors were attached to the non-dominant leg, which was defined as kicking non-preferred leg as reported elsewhere^24^. Participants were instructed to lie still on the table for 10 min in order to obtain resting values. The GXT was performed using participants’ own bicycles on an electrically braked cycling ergometer (Cyclus 2, RBM Electronics, Germany). After 5 min of warm-up at 100 W, the GXT was performed using a standard step-incremental protocol used routinely in our laboratory. Briefly, the protocol started at 100 W with step increases of 20 W.min^-1^ to volitional exhaustion^25^. Participants were instructed to maintain a steady pedal rate between 75 and 95 revolutions per minute and maintain a seated position throughout the GXT. The test was stopped when the cycling cadence dropped below 65 rpm or volitional exhaustion despite strong verbal encouragement. The peak power output (PPO) was calculated by linearly approximating the steps in the protocol and calculating the time to cessation of the test.

Gas exchange variables were measured continuously (breath-by-breath) throughout the test using Quark CPET (Cosmed, Italy) metabolic cart to determine the first (VT_1_) and second ventilatory threshold (VT_2_). Inspired and expired flow rate and gases were measured continuously and time averaged to 5-s bins. Before each test the flow rate turbine was calibrated using a 3-L calibration syringe (Cosmed, Italy), and the metabolic cart was calibrated using a known mixture within the expected ranges of O_2_ (16%) and CO_2_ (5%) concentration in accordance with the manufacturer’s recommendations (Cosmed, Italy). Heart rate was continuously monitored using a standard chest-strap heart rate monitor (H7, Polar, Finland). NIRS was measured on the vastus lateralis muscle of the non-dominant leg for measurement of muscle oxygenation values using a continuous-wave NIRS wireless sensor (Portamon, Artinis, Netherlands), utilizing six light sources with six wavelengths (759, 763, 760, 848, 844 and 848 nm) and optode distance of 35 mm. TSI was calculated using a modified Beer-Lambert law (1):

1 $$TSI= \frac{[O2Hb]}{(\left[HHb\right]+[O{2}Hb]}$$

Equation 1 TSI—tissue saturation index, O2Hb—oxygenated haemoglobin, HHb—deoxygenated haemoglobin.

In addition, relative changes of O_2_Hb, HHb and tHb were also measured and analysed.

ATT was measured on the *vastus lateralis* (VL) muscle belly using a calibrated skinfold calliper (GPM, Siber Hegner & Co., Switzerland) to ensure minimal scattering of the NIRS signal. The NIRS sensor was placed on the VL muscle belly approximately two-thirds of the distance from the anterior superior iliac spine to the lateral condyle of the femur. The probe was placed on the biggest protrusion of the VL muscle belly, after shaving and wiping the area with an alcohol solution and a clean wipe. The VL is commonly examined during cycling exercise, as VL is heavily involved in cycling exercise^5,26^. The sensor was held in place using an adhesive tape, covered by an optically dense black vinyl sheet to avoid the protrusion of light to the sensor and additionally secured in place by an elastic strap. Data were collected at a sampling frequency of 10 Hz and subsequently reduced to 5-s bins for time-aligned analysis with gas-exchange variables.

### Data analysis

The gas-exchange variables were cleaned and smoothed by removing data points with distance of > 3 SD from the local mean and linear extrapolation. The procedure was automatically performed by software (Cosmed Omnia, version: 2.2, Cosmed, Italy). The data were then time-averaged to 5 s bins. We defined VT_1_ based on the criteria for the gas exchange threshold and VT_2_ as the respiratory compensation point. Both were determined by two independent researchers with > 3 years of experience in daily GXT analysis through visual inspection and consensus according to the literature^7,27^. Briefly, VT_1_ was identified as the first inflection in the V̇CO_2_/V̇O_2_ relationship and V̇_E_/V̇O_2_ relationship. Additionally, stabilization of the end-tidal PCO_2_/V̇O_2_ and an increase in V̇_E_/V̇O_2_ versus V̇O_2_ were employed as confirmation criteria. VT_2_ was determined as a disproportionate increase in the V̇_E_/V̇O_2_ relationship and deviation from stability in the V̇_E_/V̇CO_2_ versus V̇O_2_ relationship. Additionally, a decline following a period of isocapnic buffering in end-tidal PCO_2_ was also considered for VT_2_ confirmation. VO_2peak_ was defined as the highest 30 s rolling average. If the analysis of VT_1_ and VT_2_ differed by more than 10% between researchers, another visual inspection followed until consensus was reached. Otherwise, the values were averaged before further analysis.

Similar to gas exchange measurements, aberrant NIRS values > 3 SD from the local (10 s) mean were removed before time averaging the values to 5 s bins. For further analysis and data visualization, the TSI values were normalized to resting values, defined as the average of the last 5 min of rest period to obtain values expressed as percent change from the baseline for TSI. TSI breakpoint 1 (TSI_BP1_) and breakpoint 2 (TSI_BP2_) were plotted against time and obtained using an automated segmented regression analysis script written in R studio, as suggested previously^13^. Briefly, the first 60 s of the test were ignored in order to optimize regression results due to abrupt changes in the NIRS signal at the transition from rest to exercise. The segmented regression identified the two breakpoints by minimizing the sum of square residuals to obtain the two breakpoints. Time at TSI_BP1_ and TSI_BP2_ was calculated and 25 s average of V̇O_2_ around the specified timepoint was calculated for analysis. The calculated breakpoints were analysed as oxygen uptake at the time of occurrence and percent of maximal oxygen uptake (TSI_BP1%_ and TSI_BP2%_).

The same calculation procedure was applied for HHb values to obtain HHb breakpoints (HHb_BP1_ and HHb_BP2_) by only applying one breakpoint model for segmented regression. A two-breakpoint model was also applied to detect possible breakpoints coinciding with VT_1_, depending on the individual response. HHb breakpoints were also expressed as percent of maximal oxygen uptake (HHb_BP1%_ and HHb_BP2%_).

Minimal and maximal values of NIRS-derived parameters were also analysed, taking into account the maximal normalized value achieved during the test (TSI_max_), minimal value achieved (TSI_min_), and the difference between maximal and minimal normalized values achieved during the test (TSI_diff_).

To account for variability in responses during GXT, a 4-phase model^15^ was employed as a basis for the segmented regression model. Based on the magnitude of TSI changes observed during phase 3 of the response, participants were categorized into three groups: (a) Those exhibiting a plateau or positive deflection in TSI, (b) Those showing a deflection with a lesser degree of TSI reduction compared to phase 2, (c) those exhibiting a negative deflection in TSI with a more pronounced decline compared to phase 2.

### Statistical analysis

All values are expressed as means (M) and standard deviations (SD). Students’ *t*-test was employed to test differences between groups and non-parametric tests were employed where the assumption of normality was not met. All threshold values are expressed as V̇O_2_, as gas exchange variables have been demonstrated to be independent of the rate of GXT incrementation, in contrast to the work rates at which these breakpoints occur^28,29^. A repeated measures ANOVA with post-hoc pairwise comparisons using Bonferroni correction was employed to test for differences between breakpoints obtained by different parameters. Pearson product moment correlations with 95% confidence intervals (95% CI) were computed to determine the association between breakpoints, while linear regression analysis was performed for each breakpoint pair. The following thresholds of the correlation coefficient were used to assess the magnitude of the relationships analysed: weak: ≤ 0.35; moderate: 0.36–0.67; and high: ≥ 0.68^30^. Data processing was performed in Matlab (MathWorks, version: R2024b), segmented regression was performed in R studio (version: 3) and statistical analysis was performed in SPSS software (IBM Statistics, IBM, Silicon Valley, version: 29). Statistical significance was set a priori at *p* < 0.05. Sample size was calculated using GPower software (version: 3.1.9.7) for α = 0.05 and β = 0.95 based on effect size of 0.58 (Pearson product moment correlation)^31^ for a sample size of 23. The final sample size was adjusted to obtain matched groups.

## Results

### Sample characteristics

The average PPO during GXT was 411 ± 37 W (320—467). The overall sample characteristics are presented in Table 1.

**Table 1:** Descriptive statistics

Descriptive statistics					
	N	Minimum	Maximum	Mean	Std. deviation
Age	27	16	29	21.12	4.23
Height	27	168.5	189	179.40	4.75
Body mass	27	64	90.3	73.32	6.97
BMI	27	19.5	26.8	22.78	1.84
%BF	27	4.2	18.3	10.85	3.44
SMM	27	32.5	44	37.36	3.36
VL ATT [mm]	11	4.8	10	7.52	1.69
VO_2peak_	27	52	77.7	64.00	6.29

BMI—body mass index, %BF—body fat percentage, SMM—skeletal muscle mass, VL ATT—adipose tissue thickness on the vastus lateralis muscle, VO2peak—peak oxygen uptake.

The overall sample characteristics of the EA and RA groups are presented in Table 3. There was a statistically significant difference in age (*p* = 0.006), BMI (*p* = 0.007) and VO_2peak_ (*p* < 0.001), as expected given the difference in training status of the participants.

### TSI, HHb and V̇O2 response during GXT

TSI response showed good correlation with V̇O_2_ (r = − 0.93, *p* < 0.001) and HHb values (r = − 0.99, *p* < 0.001). HHb response similarly showed good correlation with V̇O_2_ (r = 0.94, *p* < 0.001). TSI and HHb response during GXT did not show difference between EA and RA groups (Table 3). The EA group achieved lower TSI_min_ values, although the difference did not reach statistical significance (*p* = *0.081*). Correlation between TSI_min_ and VO_2peak_ was negative and moderate (r = − 0.411, *p* = 0.037).

**Fig. 1:**
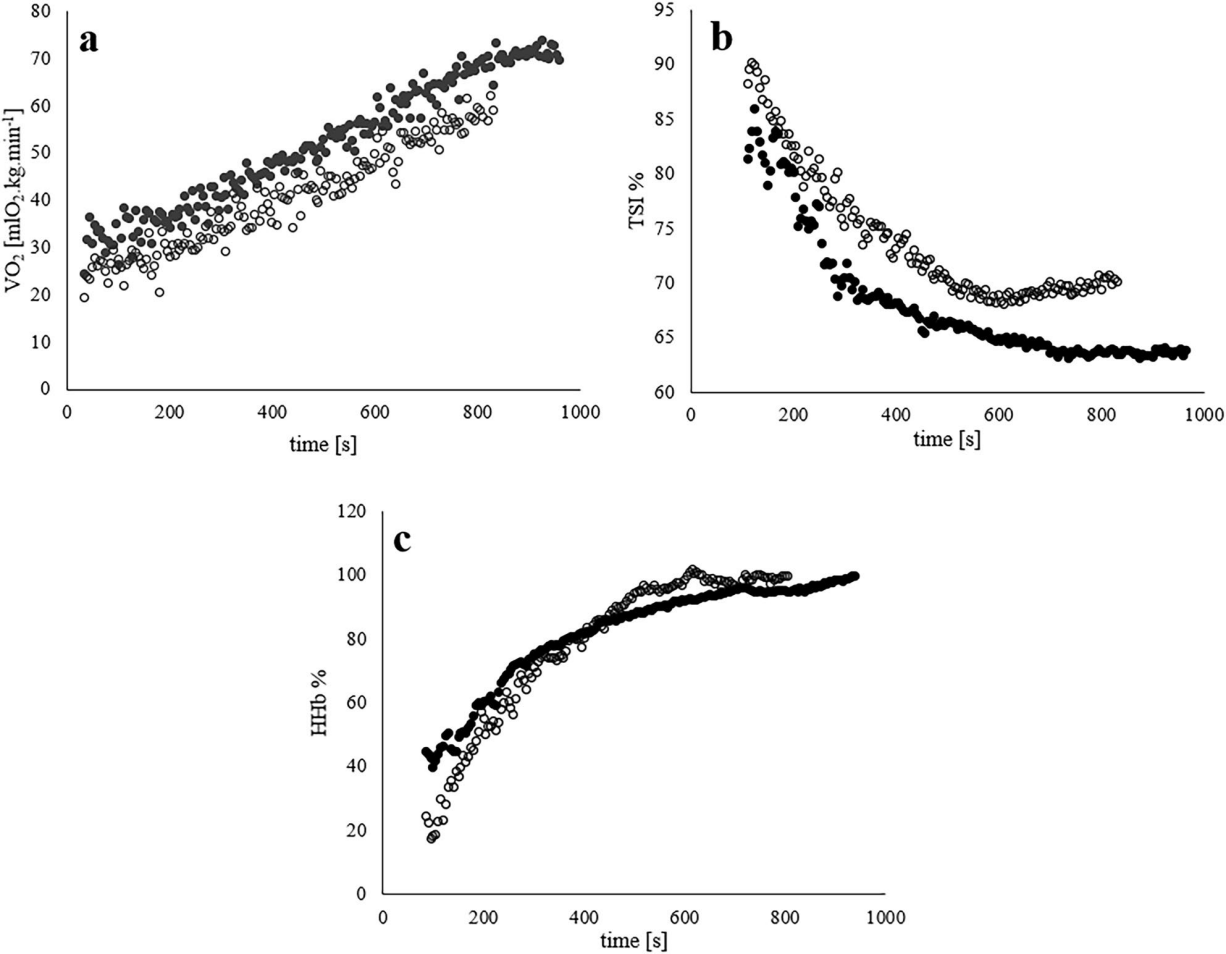
Example of response in (**a**) oxygen uptake (V̇O_2_), (**b**) TSI and (**c**) HHb% for one representative endurance and one recreational athlete during GXT. ●—endurance athlete, ○—recreational athlete.

Figure 1 represents difference in response of V̇O_2_, HHb and TSI for two representative participants from EA and RA group.

**Fig. 2:**
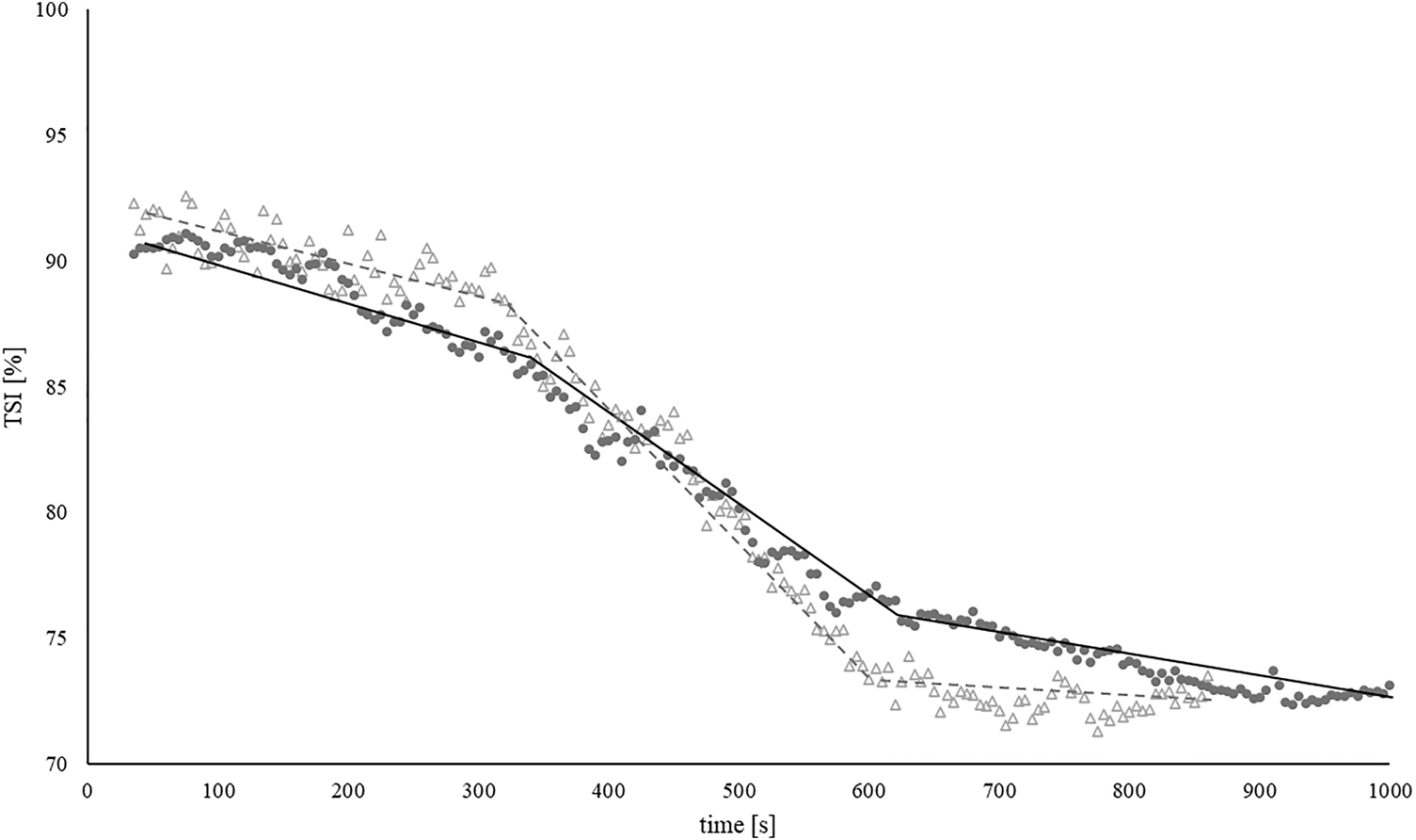
Visual representation of TSI response difference for two representative participants with linear regression analysis results. Triangles (∆)—TSI levelling off after TSI_BP2_, circles (●)—lesser degree of TSI drop after TSI_BP2_, solid and dashed lines—linear regression representation.

The time course of TSI values follows a distinct four-phase pattern^15^. After exclusion of the first 60 s of exercise to allow for stabilisation (Phase 1) the TSI values decrease steadily with increasing intensity (Phase 2), showing a distinct breakpoint in the TSI curve and continuous decline of TSI values with increasing intensity. Phase 3 represents the levelling off of TSI values until test cessation. Phase 4 of this response represents rapid reoxygenation, that was not analysed (Fig. 2).

While a levelling off during phase 3 of this response is expected, TSI values towards the end of GXT can exhibit a lesser degree of TSI drop or a second negative deflection, as visually presented in Fig. 2. A plateau in TSI values was observed in 15 participants (55%), 11 participants (45%) exhibited a lesser degree of TSI drop.

### Threshold determination and comparison

Ventilatory thresholds were calculated for all participants. TSI_BP1_ was calculated for 23 participants (85%) and TSI_BP2_ was calculated for 26 out of 27 participants (96%). After applying a one breakpoint model to HHb data, the possibility of two breakpoints was also examined. 14 participants (52%) exhibited two breakpoints in the HHb response and 11 participants exhibited only one breakpoint (41%). Two participants (7%) did not show any breakpoint in HHb response.

**Fig. 3:**
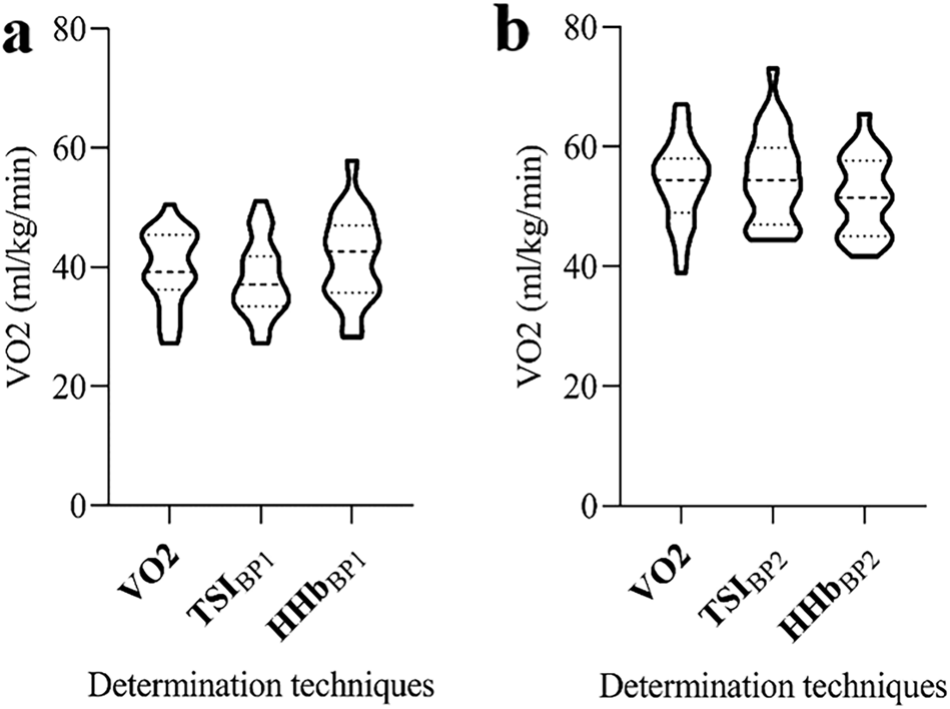
Difference in threshold determination techniques: (**a**) VT_1_, TSI_BP1_, HHB_BP1_ and (**b**) VT_2_, TSI_BP2_ and HHb_BP2_. Dashed line represents average value, dotted lines represent standard deviation.

Analysis of different thresholds on the whole sample using ANOVA with post-hoc comparison revealed no significant differences in oxygen uptake at VT_1_, TSI_BP1_ and HHb_BP1_ (F = 1.382, *p* = 0.258). Similarly, there was no difference between VT_2_, TSI_BP2_ and HHb_BP2_ (F = 1.039, *p* = 0.359). (Fig. 3).

Table 2 shows correlations between thresholds, expressed as V̇O_2_. All thresholds exhibited moderate to strong positive correlations. Pearson correlation coefficients between VT_1_, TSI_BP1_ and HHb_BP1_ ranged from 0.65—0.79 and between 0.81 and 0.9 for VT_2_, TSI_BP2_ and HHb_BP2_.

**Table 2:** Correlations between NIRS-derived variables and ventilatory thresholds obtained during incremental cycling test.

Correlations							
		VT_1_ [ml.kg.min^−1^]	TSI_BP1_ [ml.kg.min^−1^]	HHb_BP1_ [ml.kg.min^−1^]	VT_2_ [ml.kg.min^−1^]	TSI_BP2_ [ml.kg.min^−1^]	HHb_BP2_ [ml.kg.min^−1^]
VT_1_ [ml.kg.min^-1^]	Pearson Correlation	1	0.792**	0.793**	0.917**	0.788**	0.846**
	Sig. (2-tailed)		< 0.001	< 0.001	< 0.001	< 0.001	< 0.001
TSI_BP1_ [ml.kg.min^-1^]			1	0.649**	0.722**	0.770**	0.834**
				0.004	< 0.001	< 0.001	< 0.001
HHb_BP1_ [ml.kg.min^-1^]				1	0.770**	0.754**	0.836**
					< 0.001	< 0.001	< 0.001
VT_2_ [ml.kg.min^-1^]					1	0.806**	0.864**
						< 0.001	< 0.001
TSI_BP2_ [ml.kg.min^-1^]						1	0.895**
							< 0.001
HHb_BP2_ [ml.kg.min^-1^]							1

** Correlation is significant at the 0.01 level (2-tailed), VT_1_—first ventilatory threshold, TSI_BP1_—TSI breakpoint 1, HHb_BP1_—HHb breakpoint 1, VT_2_—second ventilatory threshold, TSI_BP2_—TSI breakpoint 2, HHb_BP2_—HHb breakpoint 2.

### Comparison between EA and RA

The comparison of EA and RA groups showed statistically significant differences between groups in oxygen uptake at VT_1_, TSI_BP1_, HHb_BP1_ and VT_2_, TSI_BP2_ and HHb_BP2_ (Table 2). The EA group exhibited higher oxygen uptake at all exercise thresholds compared to RA. The EA group also achieved VT_1_ and VT_2_ at higher relative oxygen uptake expressed as % VO_2peak_ compared to RA. There was no difference in relative oxygen uptake at TSI_BP1_, HHb_BP1_, TSI_BP2_ and HHb_BP2_ between groups. The EA group also achieved lower TSI_min_ values, though it did not reach statistical significance. (Table 3).

**Table 3:** Overview of the differences between highly trained endurance (EA) and recreational athletes (RA).

		Endurance athlete (EA)		Recreational athlete (RA)						95% confidence interval	
	N	Mean	SD	Mean	SD	t	Two-Sided p	Mean Difference	Cohen’s d	Lower	Upper
age	27	19.06	2.93	23.35	4.37	3.019	0.006	4.29	1.16	0.33	1.97
BMI	27	21.90	1.36	23.73	1.86	2.931	0.007	1.83	1.13	0.30	1.94
%BF	27	9.74	3.30	12.05	3.30	1.818	0.081	2.31	0.70	− 0.09	1.47
SMM	27	36.31	2.96	38.50	3.50	1.763	0.09	2.19	0.68	− 0.11	1.45
VL ATT [mm]	11	6.65	0.78	7.71	1.81	0.788	0.451	1.06	0.62	− 0.96	2.16
VO_2peak_	27	69.03	3.61	58.59	3.22	− 7.91	< .001	− 10.44	− 3.05	− 4.16	− 1.90
VLTSImax	26	97.75	5.96	99.64	4.22	0.932	0.361	1.89	0.37	− 0.41	1.14
VLTSImin	26	66.67	7.72	72.13	7.58	1.819	0.081	5.46	0.71	− 0.09	1.50
VLTSI difference	26	− 31.08	6.66	− 27.51	6.39	1.394	0.176	3.57	0.55	− 0.24	1.33
VT_1_ [ml.kg.min^−1^]	27	44.49	3.15	34.68	4.36	− 6.742	< 0.001	− 9.81	− 2.60	− 3.62	− 1.54
TSI_BP1_ [ml.kg.min^−1^]	23	42.29	5.20	33.60	3.90	− 4.5	< 0.001	− 8.69	− 1.88	− 2.86	− 0.87
HHb_BP1_ [ml.kg.min^−1^]	19	45.34	6.20	35.08	4.94	− 3.728	0.002	− 10.26	− 1.77	− 2.86	− 0.65
VT_2_ [ml.kg.min^−1^]	27	59.14	3.80	48.44	4.76	− 6.483	< .001	− 10.70	− 2.50	− 3.51	− 1.46
TSI_BP2_ [ml.kg.min^−1^]	26	60.57	5.53	49.18	4.25	− 5.888	< .001	− 11.39	− 2.31	− 3.30	− 1.29
HHb_BP2_ [ml.kg.min^−1^]	20	57.22	4.46	47.52	4.54	− 4.789	< .001	− 9.70	− 2.15	− 3.26	− 1.01
VT_1_ [%VO_2peak_]	27	64.48	3.77	59.05	5.23	− 3.116	0.005	− 5.44	− 1.20	− 2.01	− 0.37
TSI_BP1_ [%VO_2peak_]	23	61.10	7.60	52.98	17.51	− 1.475	0.154	− 8.12	− 0.60	− 1.42	0.22
HHb_BP1_ [%VO_2peak_]	19	60.31	19.89	61.14	7.14	0.106	0.917	0.83	0.05	− 0.87	0.97
VT_2_ [%VO_2peak_]	27	85.67	3.16	82.53	4.64	− 2.066	0.049	− 3.13	− 0.80	− 1.58	0.00
TSI_BP2_ [%VO_2peak_]	26	87.61	5.58	83.98	5.93	− 1.608	0.121	− 3.63	− 0.63	− 1.41	0.16
HHb_BP2_ [%VO_2peak_]	20	83.70	5.14	81.87	6.13	− 0.713	0.485	− 1.83	− 0.32	− 1.20	0.57

*BMI*: body mass index, %*BF*: body fat percentage, *SMM*: skeletal muscle mass, *VL ATT*: vastus lateralis adipose tissue thickness, VO_2peak_: peak oxygen uptake, VLTSImax: maximal TSI during graded exercise test, VLTSImin: minimal TSI during graded exercise test, VLTSI difference: difference between minimal and maximal TSI value during graded exercise test , VT_1_: first ventilatory threshold, TSI_BP1_: TSI breakpoint 1, HHb_BP1_: HHb breakpoint 1, VT_2_: second ventilatory threshold, TSI_BP2_: TSI breakpoint 2, HHb_BP2_: HHb breakpoint 2.

## Discussion

The primary aim of this study was to investigate feasibility of threshold determination using HHb and TSI during incremental cycling exercise test and compare NIRS-derived values between EA and RA cyclists. To our knowledge, this is also the first study to assess both TSI and HHb derived thresholds in highly endurance trained athletes (mean VO_2peak_ of 69.03 ml.kg^−1^.min^−1^). The main findings of this study were as follows: a) there was no difference in thresholds derived from V̇O_2_, TSI or HHb, suggesting that both TSI and HHb thresholds occurred at the same intensity as ventilatory thresholds; b) threshold determination using HHb and TSI exhibits high correlation to VT_1_ and VT_2_, and; c) EA achieved VT_1_ and VT_2_ at a higher relative intensity than RA.

While the occurrence of ventilatory thresholds is well investigated, the mechanisms behind occurrence of breaking point in HHb profile remain elusive. Studies have suggested that the mechanisms responsible for VT_2_ would result in a strong vasodilatory response in the observed tissue, resulting in the redistribution of blood flow to the working muscle^5,28^. This results in increased local oxygen delivery and dampening of oxygen extraction, causing a plateau in the HHb signal. This specific response might also be explained by muscle fiber recruitment. With increased metabolic demand, a bigger proportion of type II fibers with lower oxidative capacity is activated^32^, resulting in a plateau in the HHb signal^17^. Indeed, a plateau in HHb was observed in our study, although a significant number of participants (31%) exhibited a continuous rise of HHb values till the end of the test.

Both the method of threshold determination and data processing may play an important role in obtaining reliable results. While some studies have used only the last portion of each stage to assess HHb^33^ or TSI^34,35^ to derive thresholds, others performed analysis on a continuous scale^5,13,16–18,36,37^, similar to the method used for ventilatory threshold determination. A systematic review by Sendra-Pérez et al.^8^ has shown that the segmented linear regression is the most prevalent method for threshold determination, although several studies also used visual inspection^17,37^, D_max_ method^34^ and proprietary algorithms^38^ developed by device manufacturers. Accordingly, we used segmented linear regression on continuous data to obtain relevant breakpoints and reduce observer bias in threshold determination.

This study shows that practical estimations of VT_1_ and VT_2_ can be achieved using NIRS-derived parameters. While the criteria for estimation of VT_1_ and VT_2_ are well established, estimation of threshold using NIRS parameters is less defined and warrants further investigation. In a recent review, Sendra-Pérez et al.^8^ highlighted that some authors associate breakpoint values to VT_1_, while others link them to VT_2_. Conversely, some studies report breakpoints correlating with both VT_1_ and VT_2_^13,18,36^. We believe the difference might occur due to different threshold determination methodologies, as well as the use of different parameters to calculate thresholds. In our study we followed a two-breakpoint model, as described by Feldmann et al.^13^ to obtain both breakpoints corresponding to V̇O_2_-derived ventilatory thresholds. We were able to calculate TSI_BP1_ (86%) and TSI_BP2_ (96%) in the majority of participants, thus showing its feasibility. HHb profile during GXT has been shown to follow a sigmoidal function^10,39^, although some authors describe it as segmented linear function with stable increase and a plateau in HHb values towards the end of the test^17^. Considering this, we applied both two-breakpoint model and one breakpoint model to account for different individual responses and assess threshold determination feasibility. Results showed that approximately half of the participants exhibited two breakpoints in the HHb response, while the other half exhibited only one breakpoint in HHb response. HHb response did not show any breakpoints in two participants. Interestingly, in participants exhibiting only one breakpoint, seven HHb breakpoints coincided with VT_2_ and four with VT_1_. This difference in response may account for the variability in the results and correlation of HHb breakpoints with both VT_1_ and VT_2_ observed in the literature. In comparison to HHb, threshold determination using TSI values exhibited a more consistent response and appears to be more suitable for threshold determination in practice.

The main focus of the present study was to assess agreement between three different methods of threshold determination. We found no statistically significant difference between VT_1_, TSI_BP1_ and HHb_BP1_ as well as between VT_2_, TSI_BP2_ and HHb_BP2_. This finding suggests that both ventilatory and NIRS-derived methods of threshold determination are suitable, which is in line with existing literature^5,13,17^. Although no significant difference between HHB_BP2_ and VT_2_ was demonstrated, Iannetta et al.^17^ observed that the HHb breakpoint occurs at a slightly higher relative intensity than VT_2_ after accounting for mean response time. In the current study, HHb_BP2_ occurred slightly earlier than VT_2_. This can be attributed to different approach to analysis by accounting for mean response time, though the difference in both studies was minimal. TSI_BP2_ occurred at the same V̇O_2_ as VT_2_ in both groups. While the majority of studies comparing similar thresholds corroborate our results, Raleigh et al.^35^ observed that VT_2_ to occured after TSI_BP2_ in highly trained cyclists when looking at workload instead of V̇O_2_. Reinpõld et al.^18^ reported similar results in terms of power normalized to body mass in elite senior cyclists. Direct comparison between our findings and previous studies is challenging due to differences in data processing methodologies, step duration, and incrementation protocols. A critical distinction lies in the metrics used to define threshold values: prior studies primarily employed work rate (e.g., power output), whereas our analysis utilized oxygen uptake. This methodological divergence may account for observed discrepancies. Notably, V̇O_2_ at the VT_2_ remains consistent regardless of step duration or protocol design, as it reflects a physiological steady state. Conversely, work rate at VT_2_ is inherently influenced by the rate of load incrementation, introducing variability that complicates cross-study comparisons^7^. NIRS-derived parameters may be influenced by step duration and step incrementation in the same manner, but the consensus on this topic is currently unclear and warrants further investigation. Observed differences between ventilatory and NIRS-derived thresholds were small and can be explained by variance in both ventilatory and NIRS derived thresholds determination accuracy as well as VO_2_ data variability, which was reported to be ~ 140 mLO_2_.min^-1^ during steady state exercise^40^. Additionally, the differences might be explained by the time-delay between the occurrence of the local threshold at the level of the working muscle and the transit time needed for detection using ventilatory parameters.

We observed high correlations between different parameters when assessing both VT_1_ and VT_2_, suggesting both TSI and HHb might be a suitable proxy for non-invasive and cost-effective threshold determination. Similar to previous studies^18,36^, higher correlations were observed for VT_2_ compared to VT_1_. For example, Fontana et al.^5^ observed high correlation values between HHb_BP2_ and VT_2_. Similar to our results, Reinpõld et al.^18^ observed high correlations between TSI_BP1_ versus VT_1_ and TSI_BP2_ versus VT_2_, albeit using work rate to compare the values. Taken together, the literature demonstrates a wide range of correlations (from moderate to high) between NIRS-derived thresholds and ventilatory thresholds. This variability can be prescribed to several important factors, including differences in thresholds determination methodologies, inconsistencies in sensor placement and data processing techniques. These discrepancies make direct comparisons between studies challenging. Generally, studies using segmented regression on data recorded with high sampling rate reported values similar to that obtained in our study^16,18^. Numerous studies also compared TSI and HHb-derived breakpoints to various lactate thresholds with mixed results^35^. Even though VT_1_ and VT_2_ can be considered proxies for lactate thresholds, differences in lactate threshold determination methodology preclude direct data comparison. The participants’ training status has been shown to influence the accuracy of threshold determination^41^. This may explain notably high correlations observed in our highly trained sample compared to studies with less trained or heterogeneous cohorts.

A clear difference in maximal oxygen uptake and maximal power output was observed between EA and RA group. The lowest attained TSI (TSI_min_), which reflects localized oxygen extraction capacity, was correlated with maximal oxygen uptake^13^. Although not statistically significant, the EA group exhibited lower TSI_min_ values compared to the RA group. In addition to differences in TSI_min_ between the groups, a moderate correlation (r = − 0.411, *p* = 0.037) was observed between VO_2peak_ and TSI_min_ across the entire sample, further suggesting a trend toward enhanced peripheral oxygen utilization along with higher systemic maximal oxygen uptake. There was no difference in other variables, such as TSI_max_ and TSI_diff_. Notably, the EA group demonstrated higher relative oxygen uptake at both thresholds compared to the RA group. These findings align with characteristics of highly trained athletes, who typically exhibit greater systemic VO_2max_, ability to sustain higher work rate even with the significantly lower tissue oxygenation (indicative of efficient oxygen extraction), and elevated oxygen uptake at metabolic thresholds^42^. Such insights could assist coaches in evaluating athletes’ trainability and tailoring interventions to optimize physiological adaptations. Future studies aimed to investigate the physiological mechanisms underlying the observed trends in TSI_min_ (e.g., microvascular density, mitochondrial efficiency, or muscle fiber type) to clarify how localized oxygen extraction influences systemic VO_2max_ and threshold performance are needed. Additionally, it would be beneficial to explore whether the relationship between TSI_min_ and VO_2max_ varies across athlete populations (e.g., endurance vs. strength athletes) or training stages (e.g., novice vs. elite).

Although the present study has many unique strengths, such as the inclusion of a highly trained athletes and the comparison between less- and highly trained individuals, certain limitations should be acknowledged. First, we were unable to perform a physiological calibration for the NIRS parameters. While this precluded us to report changes in HHb concentration, it did not affect the linear regression analysis and consequently identification of NIRS-derived thresholds. In fact, this approach could be considered a strength as the method used is more practical for athletes and coaches and therefore has greater applicability in real-world settings. Second, we did not calculate the mean response time, which prevented us from expressing threshold comparisons in terms of power output. We advise that future studies address these methodological aspects and report selected thresholds in both power and oxygen uptake terms to improve interpretability and practical application.

## Conclusion

This study found no significant differences in VT_1_ and VT_2_ determination when using TSI, HHb and V̇O_2_ parameters. Additionally, a high correlation was observed between ventilatory thresholds and NIRS-derived thresholds, using both HHb and TSI as parameters. Among the examined parameters, TSI exhibited lower response variability compared to HHb for determination of both metabolic thresholds, making it more suitable for analysis and practical applications. While the methodology for ventilatory thresholds is well established, the same can’t be said for TSI and HHb-based threshold determination. Although multiple approaches exist for identifying NIRS-derived thresholds, we encourage using segmented regression using time bins similar to those commonly applied for ventilatory threshold determination. Aligning these methodologies would enhance consistency between NIRS- and ventilatory-based approaches. Furthermore, incorporating the automatic or semi-automatic methods for threshold determination can help minimize observer bias and improve the reproducibility of future studies. Not only do highly trained cyclists achieve higher maximal oxygen uptake, but also demonstrate lower minimal TSI values compared to their recreationally trained counterparts. This suggests superior peripheral oxygen extraction capabilities in the highly trained group.

Practical implications:

- NIRS-derived parameters can be used reliably to assess exercise thresholds
- There is a difference in muscle oxygenation between highly trained and recreative cyclists
- NIRS can be used to complement ventilatory parameters in exercise testing

## Data Availability

The datasets generated and analysed during the current study are available from the corresponding author upon reasonable request.
